# Changes in Carotenoids and Quality Parameters of Sweet Paprika (*Capsicum annuum*) After an Accelerated Heat Treatment

**DOI:** 10.3390/antiox13121492

**Published:** 2024-12-06

**Authors:** Belén Olga Ferrando, Nieves Baenas, María Jesús Periago

**Affiliations:** Department of Food Technology, Food Science and Nutrition, University of Murcia, Campus of International Excellence “Campus Mare Nostrum”, 30100 Murcia, Spain; obelen.ferrando@um.es (B.O.F.); mjperi@um.es (M.J.P.)

**Keywords:** temperature, β-carotene, esterified carotenoids, antioxidant capacity, CIELAB, ASTA, HPLC-DAD

## Abstract

Paprika, obtained from dried red pepper (*C. annuum*), is valued for its characteristic colour and flavour. Its carotenoid content, which is important for both sensory and nutritional quality, varies depending on several factors (agronomic conditions and technological treatment with special attention to the drying methods) that influence the colour and antioxidant capacity of the samples. This study investigated the effect of an accelerated thermal treatment (80 °C for 8 h) applied to evaluate the oxidative stability of the carotenoids and the colour of ground paprika depending on its origin (Peru or China). Changes in quality parameters (ASTA index and CIELAB colour), carotenoid content and profile (HPLC-DAD) and lipophilic antioxidant capacity (FRAP and ABTS^•+^ methods) were evaluated. Untreated Chinese samples had 30% more total carotenoids than Peruvian samples, but this was not reflected in ASTA units, indicating that at least a 50% carotenoid variation is required for significant differences. Treatment resulted in a carotenoid loss of 70% in Peruvian and 30% in Chinese samples, with changes in total carotenoids correlating positively with antioxidant capacity. Both origins had similar carotenoid profiles, with β-carotene being the predominant carotenoid, while distinct contents were observed between the origins. The higher content of esterified carotenoids in Chinese peppers resulted in better thermal stability. The results highlight the necessity for tailored production processes to maintain nutritional integrity and antioxidant capacity.

## 1. Introduction

Among the most widely consumed spices, paprika, derived from the dried ground pepper (*Capsicum annuum*) of the *Solanaceae* family, can be either sweet or hot—depending on its origin—and has attracted great attention because of its organoleptic properties. The fact that one of its main characteristics is its bright red colour means that it is widely used in the food industry as a colouring agent [1], as well as having a very distinctive flavour and taste, making it a safe alternative to the use of synthetic colours, even in cosmetic and pharmaceutical products [2]. Beyond its role as a colouring or flavouring agent, paprika has potential health benefits due to its contents in bioactive compounds. It is a rich source of carotenes (mainly α-carotene and β-carotene), xanthophylls (primarily violaxanthin, capsanthin, capsorubin, and zeaxanthin) and (poly)phenols, while it also contains essential vitamins (such as vitamins C and E) and minerals [3,4].

Carotenoids are responsible for the colour quality parameters of paprika, and within the same species, their content may vary depending on the pepper variety [5] and environmental conditions during growing (such as temperature, light intensity and soil composition) and ripening stage, which influence the biosynthesis and accumulation of these bioactive compounds during plant growth [6]. The amount of carotenoids in paprika is influenced by the pre- and post-harvest techniques used; therefore, the quality of the final product varies depending on the production process [7,8]. These vegetables are harvested when they reach their optimum point of ripeness, displaying intense colour, which indicates a high carotenoid content and low water content. In red peppers, carotenoids exist in free form as well as in monoesters and diesters bearing different fatty acid moieties; the fatty acid moiety is expected to vary, and this change in structure determines both their stability throughout processing and storage, as well as their bioavailability. Theoretically, esterification has a protective effect, which is why they are more heat stable than their counterparts [9]; however, this effect depends on the lipid moiety since the unsaturated fatty acid esterified carotenoids promote oxidation by the propagation of the radical chain [10]. In addition, it is not yet clear, but the chemical form of esterified carotenoids has the potential to improve their bioavailability [11].

The technological process of paprika may vary depending on the region and the traditional methods used, but in general, it involves selecting the peppers or raw materials, washing to remove dirty and contaminants, open-air or mechanical drying (hot air, microwave, infrared, and freeze drying), followed by grinding to obtain a fine powder. Optionally, a heat treatment is applied to sterilise/pasteurise the product with the aim of preventing mould growth and toxification during storage. The drying methods applied during processing are important to preserve the quality characteristics of paprika, particularly the colour parameters and the carotenoid content [12]. For this reason, colour and carotenoid losses during drying have been extensively studied [13,14], and it is known that the degradation of natural pigments is proportional to the drying temperature and time and fits well with both Weibull and first-order models [15].

After obtaining the paprika, its quality is determined by the absence of mycotoxins and aflatoxins and its organoleptic properties (colour intensity, aroma and flavour), being the colour, commonly measured by the American Spice Trade Association (ASTA value), the most important parameter to determine its quality and price on the market [16]. Colour is positively correlated with the content of natural pigments or carotenoids; hence, the colour losses during processing lead to a reduction of the nutritional value of paprika. Its consumption contributes to the intake of vitamin A due to the provitamin A activity of some carotenoids and also exerts a beneficial effect on human health associated with the potential prevention effect against cardiovascular diseases, neurodegenerative disorders (such as Alzheimer’s or Parkinson’s), some types of cancer and certain chronic diseases (type II diabetes) [17,18,19].

As mentioned above, the scientific literature mainly focuses on the analysis of colour changes (using the ASTA value) and carotenoid degradation during drying [20,21]; however, scarce information is available about the changes in the quality parameters of paprika during shelf life. Colour degradation and carotenoid losses continue to occur during storage, mainly due to the presence of enzymes, microorganisms, moisture, light exposition, or inappropriate temperature conditions, which lead to oxidation reactions, particularly the degradation of carotenoids [10,22]. The accelerated heat treatment can be used to evaluate the degradation and oxidation of pigments in carotenoid-rich products [23], followed by the analysis of ASTA values, carotenoid content and antioxidant capacity. These studies provide information to manufacturers and importers about the paprika quality changes and assess the nutritional quality and the potential beneficial effects for human health during shelf life.

Paprika is produced and consumed globally. According to the Food and Agriculture Organization of the United Nations [24], approximately 37 million tonnes of peppers were produced worldwide in 2022, with China, Spain and Peru among the main producers, producing 16.8, 1.5, and 0.2 million tonnes, respectively. The yield of paprika production is less than 20% of pepper production (around 5 to 6 kg of pepper are used to produce 1 kg of paprika) [25]. Besides, Spanish domestic production is insufficient to meet the high demand for this spice, and it is necessary to import it from third countries, which determines the final quality parameters of paprika. Considering that many factors can influence the stability of the quality parameters during shelf life, the origin is an important variable to take into consideration with the aim of choosing raw materials with the best quality. Thus, this work investigated the effects of an accelerated thermal oxidation experiment (80 °C for 8 h ) on the main quality parameters of sweet paprika from Peru and China, oxidative stability, colour changes (ASTA index and CIE-Lab colour coordinates), total and individual carotenoid content (analysed by HPLC-DAD) and lipophilic antioxidant capacity (evaluated by the Ferric Reducing Antioxidant Power and ABTS^•+^ cation reduction capacity methods).

## 2. Materials and Methods

### 2.1. Reagents and Chemicals

Carotenoid standard (β-carotene) was purchased from LGC Standard (Barcelona, Spain). Methanol (MeOH), acetone, hexane, tert-butyl methyl ether (TBME), and acetonitrile (ACN), all of them with HPLC grade, ethanol and sodium chloride, were purchased from Honeywell (Seelze, Germany). The 2,2′-azino-bis (3-ethyl-benzothiazoline-6-sulfonic acid) diammonium salt (ABTS^•+^), 2,4,6-tripyridyl-s-triazine (TPTZ), iron (III) chloride hexahydrate, manganese (IV) oxide, 2-propanol and the standard 6-hydroxy-2,5,7,8-tetramethylchroman-2-carboxylic acid (trolox) were obtained from Sigma-Aldrich (Steinheim, Germany). Formic acid, hydrochloric acid (37%), monopotassium phosphate, sodium acetate, and ammonium acetate were purchased from Panreac Quimica SA (Castellar del Vallés, Barcelona, Spain). Ultrapure water was produced using a Millipore water purification system (Barbataín, Spain).

### 2.2. Plant Material and Accelerated Thermal Oxidation Treatment

The samples used in the present study were sweet paprika supplied by a multinational company located in Murcia, Spain. A total of 30 paprika samples from two origins (15 from Peru and 15 from China) were used, each sample corresponding to one grind, selected based on colour units (ASTA value), ranging from 150 to 200 ASTA units. Two 50 g portions were taken from each paprika grind (sample) for the purpose of the study. One portion was subjected to heat accelerated treatment, while the other was analysed without such treatment. To study the stability of colour parameters and carotenoids during storage, the selected conditions were 80 °C for 8 h, following the assay described by Abbeddou et al. [23] with some modifications. These authors evaluated the impact of the heat treatment at 80 °C/48 h on the degradation of colouring capacity, carotenoid content, and antioxidant activity of the tomato and paprika oleoresins. Both treated and untreated portions were then stored in the dark and protected from moisture, pending analysis of the quality parameters, with each sample analysed in triplicate.

### 2.3. Carotenoids Extraction

For the carotenoids extraction, 50 mg of sample and 20 mL hexane:acetone:ethanol (2:1:1; *v:v:v*) were mixed in a vortex (VM-10, WiseMix, Wertheim, Germany). The mixtures were sonicated (5510, Branson, Danbury, CT, USA) for 2 min, and 2 mL of water was added. The carotenoids were extracted in the nonpolar phase [26].

### 2.4. Analysis of Individual Carotenoids by HPLC-DAD

For the identification and quantification of carotenoids by high performance liquid chromatography with a diode array detector (HPLC-DAD), 1 mL of the nonpolar phase was evaporated in the speed vacuum concentrator (Concentrator plus, Eppendorf, Germany), then, 1 mL of MeOH:TBME (1:1, *v:v*) was added to the sample, sonicated for a few seconds, and finally, injected into the HPLC-DAD system (Agilent 1200, Agilent Technologies, Madrid, Spain) fitted with a quaternary pump, a degasser, a thermostatic column support, an autosampler, and a serial diode detector. The identification and quantification of carotenoids by chromatographic techniques were performed following the method described by Böhn (2001) [27] using a C30 column 250 mm × 4.6 mm, 5 μm i.d. (Análisis Vínicos S.L., Villarobledo, Spain) maintained at 17 °C; TBME (A) and MeOH (B) were used as mobile phases at a flow rate of 1 mL/min. The gradient started with 2% A in B to reach 35% A at 35 min, 60% A at 45 min, 60% A at 55 min, and back to the initial conditions (2% A in B) for 5 min to stabilise the column before the next injection. The identification and quantification of free carotenoids were achieved by chromatographic comparisons of their UV spectra and retention times with the authentic standard available within the research group and those described in the scientific literature [28,29,30,31]. The unidentified carotenoids α-carotene and β-carotene were quantified using the calibration curve of β-carotene (ranging from 1.63 to 32.66 ng/µL; LOQ: 0.54 ng/µL; LOD: 0.08 ng/µL; r^2^ = 0.9987). In our research group, we do not have standards for carotenoid esters; they were tentatively identified based on their retention times and spectral characteristics described in the literature (with a maximum at 450 and 478 nm), which revealed that no alterations to the carotenoid molecule chromophore occur as a result of acylation with fatty acids [32,33,34]. Carotenoids were quantified at 450 nm. Results were expressed as mg carotenoids/g of paprika.

### 2.5. Lipophilic Antioxidant Capacity

The antioxidant capacity of the nonpolar phase containing carotenoids present in the samples was analysed by two different spectrophotometric methods: Ferric Reducing Antioxidant Power (FRAP) and ABTS^•+^ cation reduction capacity, following the procedures described by Benzie & Strain [35] and Miller et al. [36], respectively. Briefly, in the FRAP assay, 1 mL of FRAP and 300 µL of sample were mixed for 4 min in a vortex. After resting for 1 min, the absorbance was measured in the spectrophotometer (Evolution 300, Thermo Scientific, Altrincham, UK) at 593 nm, using FRAP reactive as blank. For the ABTS^•+^ assay, 1 mL of ABTS^•+^ and 100 µL of sample were vortexed for 30 s. After 50 min rest in darkness, the samples were centrifuged (5804 R; Eppendorf, Germany), and absorbance was measured in the spectrophotometer (Evolution 300, Thermo Scientific, UK) at 734 nm. Trolox was used as an antioxidant standard in both methods.

### 2.6. CIELab Colour Determination

A Chroma Meter CR300 reflectometer (Konica-Minolta, Chiyoda, Japan) was used to measure the colour coordinates, obtaining the colour coordinates of the CIEL* a* b* space, colour saturation (metric chroma, C*) and the metric hue angle (H°). In the CIEL* a* b* system, L* represents lightness, ranging from 0 (black) to 100 (white); a* represents the reddish and greenish tones, with positive values representing reddish colours and negative values representing greenish colours; b* represents yellow (positive) to blue (negative); C* represents saturation or intensity. The Hue angle (H°) is also related to the red colour, as it represents the maximum redness at the +a* axis (0 °), and it is increased to yellowness (90 °), greenness (180 °) and blueness (270 °). The paprika powder samples were measured directly using a CR-A50 glass cuvette.

### 2.7. ASTA Colour Determination

To determine the extractable colour value (American Spice Trade Association, ASTA) of the samples, 4 g of sample was weighed and volumetrically tested in 100 mL of 100% acetone. The samples were then allowed to stand at least 16 h in the dark at room temperature (RT). Then, 5 mL was removed and made up to 50 mL with acetone in a volumetric flask, and the absorbance was measured. Spectrophotometric measurements were performed at 460 nm (Zuzi 4251/50, Auxilab, Navarra, Spain) in the UV-Vis range using 1.0 cm glass cuvettes, with acetone as blank.

The calculation of colour in ASTA units was performed using the equation:

$$ASTA\;units\;=\;\frac{Abs\times 16.4\times IF}{Sample\;weigth\;(g)}$$

where *Abs* is the absorbance of the acetone extract at a wavelength of 460 nm and

*IF* = 10 is the instrumental correction factor.

### 2.8. Statistical Analyses

The statistical analysis was performed with the SPSS program (version 28.0, IMB). The results for each of the parameters analysed were expressed as the mean value ± standard deviation of three determinations. A two-way ANOVA was carried out to test whether there was an interaction between the origin of the peppers and the heat treatment applied. The data were tested for the normality of the distribution (Shapiro–Wilk) and subjected to Levene tests to evaluate the homogeneity of the variance, using an unpaired t-test for normal distribution data or a Mann–Whitney-U test for nonparametric test data, with a significance level of 95% (*p* < 0.05).

## 3. Results and Discussion

### 3.1. Influence of Origin and Thermal Treatment by Two-Way ANOVA

A two-way ANOVA was performed to ascertain the influence of the origin (O), thermal treatment (T), and the interaction between these two factors (O × T) on the different parameters analysed in the samples of sweet paprika (Table 1). The origin exerted a considerable influence (*p* < 0.001) on the content of total and individual carotenoids, as well as on the antioxidant capacity measured by FRAP. Regarding the colour parameters, only the L* and H° angles were significantly affected by this factor (*p* < 0.01). The thermal oxidation treatment also showed a strong significant effect (*p* < 0.01) on all dependent variables except for coordinates L* and a*. However, most of the analysed variables were not affected by the interaction of both factors (O × T), with only the red colour (a*) and the antioxidant capacity by the FRAP method showing statistical significance.

In summary, our results indicated that the origin of the peppers and the heat treatment applied to the paprika exhibited an important effect on the quality parameters of paprika independently. The origin can significantly influence the final quality of the samples due to factors such as climate, agricultural practices and processing techniques. Furthermore, heat treatment can result in a loss of bioactive compounds. These factors were found to determine aspects such as flavour, colour, nutrients, and bioactive compound levels [37,38,39].

### 3.2. Carotenoids

**Figure 1:**
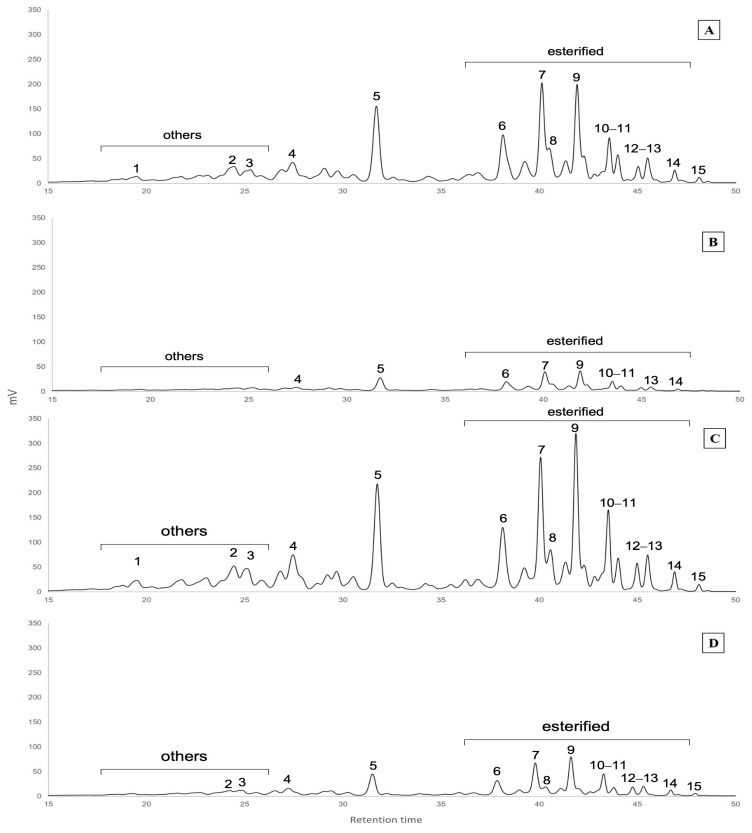
Chromatograms of the samples from untreated Peru (**A**), treated Peru (**B**), untreated China (**C**), and treated China (**D**). The observed peaks include those of other carotenoids (peaks 1, 2, and 3), α-carotene (4), β-carotene (5), and esterified carotenoids (peaks 6, 7, 8, 9, 10, 11, 12, 13, 14, and 15).

After analysing the carotenoid content in sweet paprika of Chinese and Peruvian origin in both untreated and thermal treated samples, it was observed that the carotenoid profile was similar in all the samples. This is shown in Figure 1, which revealed that the main identified carotenoids were β-carotene (peak 5), followed by α-carotene (peak 4). Furthermore, additional peaks were observed, representing unidentified carotenoids (peaks 1–3) and esterified carotenoids (peaks 6–15).

**Figure 2:**
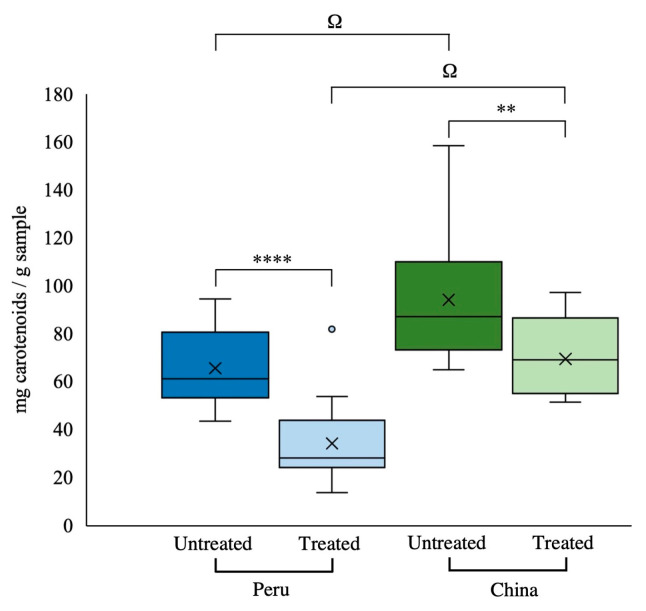
Content of total carotenoids of Peruvian (blue) and Chinese (green) origin. Untreated (intense colour) and treated (light colour). ** Indicates significant differences between samples within the same origin; ** (*p* < 0.01), **** (*p* < 0.001). ^Ω^ Indicates a significant difference between samples from different origins and the same setpoint (untreated or treated) (*p* < 0.001).

Following quantification, the results revealed significant differences in the total and individual concentrations of carotenoids according to the origin of the samples (Table 2 and Figure 2). Figure 2 shows the variability in the content of total carotenoids in untreated samples, with mean values of 66 and 94 mg/g of sample in Peruvian and Chinese paprika, respectively.

The individual carotenoids present in the samples are shown in Table 2. The untreated samples exhibited β-carotene contents of 20.7 and 23.9 mg/g of sample in Peruvian and Chinese paprika, respectively, with no significant differences observed. However, α-carotene was quantified in all samples with mean values of 12.2 and 21 mg/g, showing statistically significant differences according to the origin. The observed variations in the carotenoid profile may be attributed to the agronomic conditions during cultivation in the country of origin, which influence the final fruit characteristics and composition [37]. In addition, it is known that the carotenoid profile of paprika varies greatly depending on the variety of pepper [1,38]. For instance, some chilli peppers have been found to contain major carotenoids capsanthin and zeaxanthin, while other chillies mainly contain lutein and β-carotene [3,29,40]. There are few publications that characterise carotenoids in sweet peppers for paprika production, which are distinct from spicy peppers or chilli peppers. Our results are in agreement with those reported by Gola et al. [41], who showed β-carotene as the predominant carotenoid of paprika, while other authors have reported high contents of lycopene [42].

According to the literature, many esterified carotenoids occur in common peaks in chromatographic analysis, which prevents accurate identification. Esterified carotenoids have been tentatively identified on the basis of their characteristic spectra (with maxima at 451 and 478 nm) and retention times according to the literature (peaks 6 to 15 in Figure 1) [28,29,30]. We quantified 11 esterified carotenoids, representing approximately between 25 and 30% of the total carotenoids, with significantly different mean values of 18 and 28.3 mg/g of untreated samples of Peru and China paprika, respectively (Table 2).

In plant foods, the content of esterified carotenoids depends on the stage of ripeness at which the fruit is harvested [43]. Esterification can be influenced by environmental conditions and promoted by enzymatic activity during vegetable storage [44]. The development of esterification is linked to the ripening of the fruit, the degradation of chlorophylls, and the change from chloroplasts to chromoplasts. The degree of esterification (total, partial or absent) depends on the number of OH groups susceptible to esterification [45]. For this reason, xanthophylls (such as β-cryptoxanthin, capsanthin, lutein, and zeaxanthin) in *C. annuum* bind to fatty acids, frequently forming more stable mono or diesterified carotenoids, with lauric, myristic and palmitic acids representing the major fatty acids involved in this process [30,33,34]. In this study, these xanthophylls were not found in the free form, suggesting that they may be present in their esterified form. This is in accordance with Souza et al., who showed that the concentrations of xanthophylls in their free form were significantly lower than those in their esterified form [45]. The newly formed molecules are more resistant to thermal degradation than their free counterparts [46,47]. Regarding their physicochemical properties, esterified compounds formed by combining a carotenoid with one or more fatty acids have shown greater solubility than non-esterified carotenoids [48], increasing their bioavailability and potential beneficial effects for human health [11,49].

Additional peaks were observed and quantified as “other carotenoids” based on their characteristic UV spectrum (peaks 1, 2, and 3, with a maximum at 444 and 466 nm; Figure 1). However, their identification was not possible, as no compounds similar to those retention times were found in the existing literature. The content of “other carotenoids” for the untreated samples also showed significant differences according to the origin of the paprika samples (Table 2).

In the present study, an accelerated thermal treatment was applied to evaluate the antioxidant and carotenoid stability and the colour of paprika, with consideration of the influence of the origin. The application of heat treatments to plant tissues results in cellular degradation, which can ultimately lead to cell and tissue collapse [50]. This cellular damage leaves the interior contents unprotected against external agents, rendering them more susceptible to degradation. Following exposure to thermal treatment, carotenoids may undergo degradation or isomerisation [51]. Our results showed that the application of heat treatment may cause both transformations of carotenoids. This was evidenced by the disappearance of certain carotenoids (peaks 1 to 3, 12, and 15) in the chromatogram of the heat-treated samples, which indicates isomerisation, while others exhibited a reduction in their intensity, which suggests degradation (peaks 4 to 11, 13, and 14) (Figure 1).

The effect of the thermal treatment on total carotenoids is shown in Figure 2. As mentioned above, in both untreated and treated samples, a higher concentration of total carotenoids was quantified in the Chinese samples (94.3 and 69.6 mg/g, respectively) compared to the Peruvian samples (65.7 and 34.4 mg/g, respectively). The application of the accelerated thermal oxidation treatment led to a significant reduction of the total and the individual carotenoids in the samples, with Peruvian samples showing a more pronounced decline, with a 48% decrease in total carotenoids, compared to the 27% reduction observed in Chinese paprika samples (Figure 2).

Our results demonstrated that Chinese paprika showed not only a higher carotenoid content but also greater stability during the heat treatment; thus, it would be expected to have more stability during the commercial shelf life. Topuz et al. [52] studied the effect of different drying methods (refractance window drying, freeze-drying, oven-drying, and natural convective drying) on the carotenoid and capsaicinoid content of paprika *cv. Jalapeno*, reporting that the more aggressive the heat treatment, the greater the loss of these bioactive compounds. Schweiggert et al. [39] studied the stability of carotenoids (esterified and non-esterified) in paprika and showed that heat treatments at 80, 90, and 100 °C for 5 and 10 min were sufficient to reduce the carotenoid content by 20 to 53%. Consequently, the losses observed in this research would be expected within this range.

The stability of the carotenoids to oxidation also depends on the chemical structure since the degree of esterification also influences thermal stability, with diesters being more resistant to degradation than monoesters [16,35]. The fact that they are attached to one or more fat molecules seems to give them a higher oxidative and thermal stability [30,53]. Nevertheless, there is a certain degree of controversy within the scientific community with regard to the oxidative stability of esterified carotenoids. Some authors claim that the binding of carotenoids to these fat molecules gives them a certain resistance to stressors such as light, storage time or temperature [10]. However, other authors claim that carotenoids bound to polyunsaturated fatty acids have a lower oxidative stability than their free form, as these acids provide a more oxidative environment, generating more reactive species and leading to an accelerated degradation of the compound [54]. Our results indicated that the proportion of esterified carotenoids was slightly higher in Chinese paprika samples (41%) than in Peruvian samples (37%), which could lead to a reduction in the degradation of total carotenoids.

### 3.3. Lipophilic Antioxidant Capacity

Paprika is a rich source of carotenoids, which are recognised for their antioxidant properties. As described above, it is expected that a decrease in these bioactive compounds might lead directly to a reduction of this capacity. To ascertain the lipophilic antioxidant capacity of paprika samples, two complementary spectrophotometric methods were used: FRAP measures the ability of the compounds to transfer electrons and reduce ferric iron (Fe^+2^) to ferrous complex (Fe^+3^), whereas ABTS^•+^ is based on the neutralising capacity of the cation through the transfer of electrons and hydrogen atoms from the sample [55]. Both methods are applicable to evaluate the antioxidant capacity of lipophilic compounds, showing a strong correlation among them. Carotenoids, in particular, have been described as able to transfer electrons and hydrogen atoms [56]. The present study revealed a positive correlation between total carotenoid content and antioxidant activity, as determined by the FRAP and ABTS^•+^ methods, in samples from Peru (r = 0.567 and r = 0.656, *p* < 0.01, respectively) and China (r = 0.637, *p* < 0.01 and r = 0.39, *p* = 0.08). Besides, FRAP and ABTS^•+^ values in all samples from China and Peru showed a strong correlation (0.714 and 0.885, *p* < 0.01, respectively). Regarding the effect of thermal treatment, no correlation was found between carotenoid content and antioxidant capacity, which could be explained by the degradation of carotenoids.

**Figure 3:**
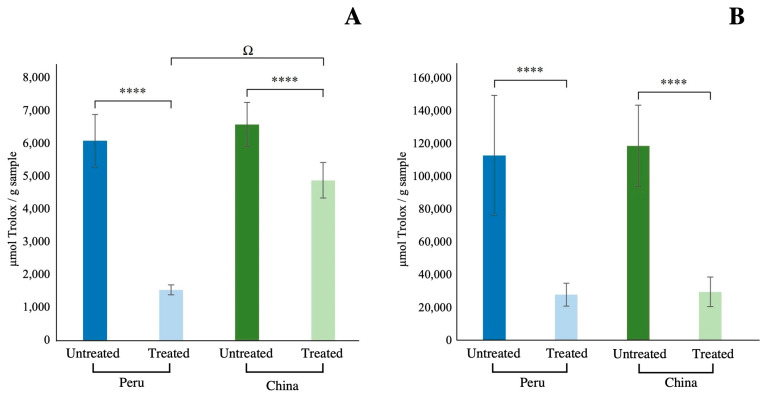
Lipophilic antioxidant capacity expressed as mmol Trolox/g sample, FRAP (**A**) and ABTS^•+^ (**B**) of Peruvian (blue) and Chinese (green) origin. Untreated (more intense colour) and treated (lighter colour). **** Indicates significant differences between samples within the same origin (*p* < 0.001). ^Ω^ Indicates a significant difference between samples from different origins and the same set point (BHT or AHT) (*p* < 0.0001).

The results of antioxidant capacity showed no statistically significant differences between the untreated samples from both origins. The mean values for FRAP and ABTS^•+^ were 6.6 and 117.9 mmol Trolox/g for the Chinese samples and 6.1 and 112.2 mmol Trolox/g for the Peruvian paprika, respectively (Figure 3). The 30% greater carotenoid content in the untreated samples from China in comparison to Peru did not result in a greater antioxidant capacity in the samples, but it is known that paprika also contains other antioxidant compounds, such as (poly)phenols, that could contribute to the FRAP and ABTS^•+^ values [12,42].

However, after thermal treatment, significant differences (*p* < 0.0001) were observed within the same origin for both antioxidant capacity assays (Figure 3). The comparison of heat-treated samples from different origins revealed significant differences in the FRAP assay (*p* < 0.0001). The FRAP values of the Chinese samples were less affected by heat treatment than those of the Peruvian samples, which could be due to the presence of esterified carotenoids in the former but also to the degree of saturation of the fatty acid to which they are attached [57]. Moreover, the application of high temperatures to plant tissues can result in the disruption of the cell wall [50], which, together with the chopping that occurs during production, may lead to the release of additional antioxidants (vitamins and phenolic compounds) that can potentially contribute to the prevention of the oxidative degradation of carotenoids [38]. In this study, the lipophilic antioxidant capacity was only evaluated, but in addition to carotenoids, other lipophilic compounds may be present in Chinese paprika, contributing to the antioxidant properties. These may include lipophilic phenolic compounds. Bouziane-Ait Bessai et al. [58] showed that the antioxidant capacity of paprika also depends on the storage conditions (temperature and time); they observed that after 6 months, samples stored frozen had almost twice the antioxidant capacity of those stored at room temperature.

The carotenoids present in paprika have been associated with a number of beneficial effects, including antioxidant, chemopreventive, anti-tumour, skin photoprotective, anti-inflammatory or anti-diabetic activity [59,60]. Regular consumption of this spice is considered an effective way to achieve the recommended intake of carotenoids (5 to 10 mg/day for β-carotene) and obtain their associated health beneficial effects [61]. Although both the total carotenoid content and the lipophilic antioxidant capacity were significantly reduced after the oxidation thermal treatment, these parameters might be taken into consideration in the shelf life studies to ensure the provision of products with optimal nutritional and beneficial effects to consumers.

### 3.4. Colour

**Figure 4:**
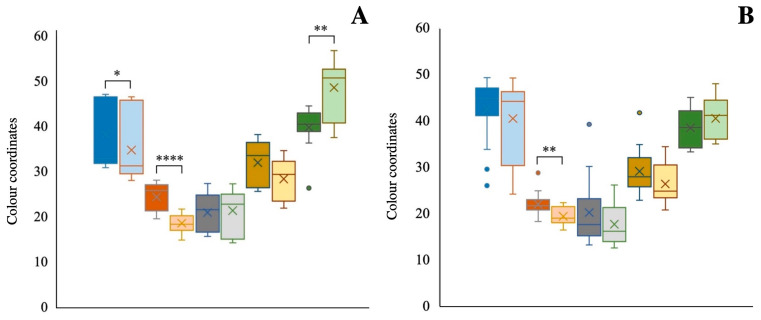
Coordinates CIE L*, a*, b*, C* and H° in samples of Peruvian (**A**) and Chinese (**B**) origin. L* (blue), a* (orange), b* (grey), C* (yellow), and H° (green) colour coordinates ordered from left to right, respectively, untreated (more intense colour) and treated (lighter colour). * Indicates significant differences between samples within the same origin; * (*p* < 0.05), ** (*p* < 0.01), **** (*p* < 0.0001).

**Figure 5:**
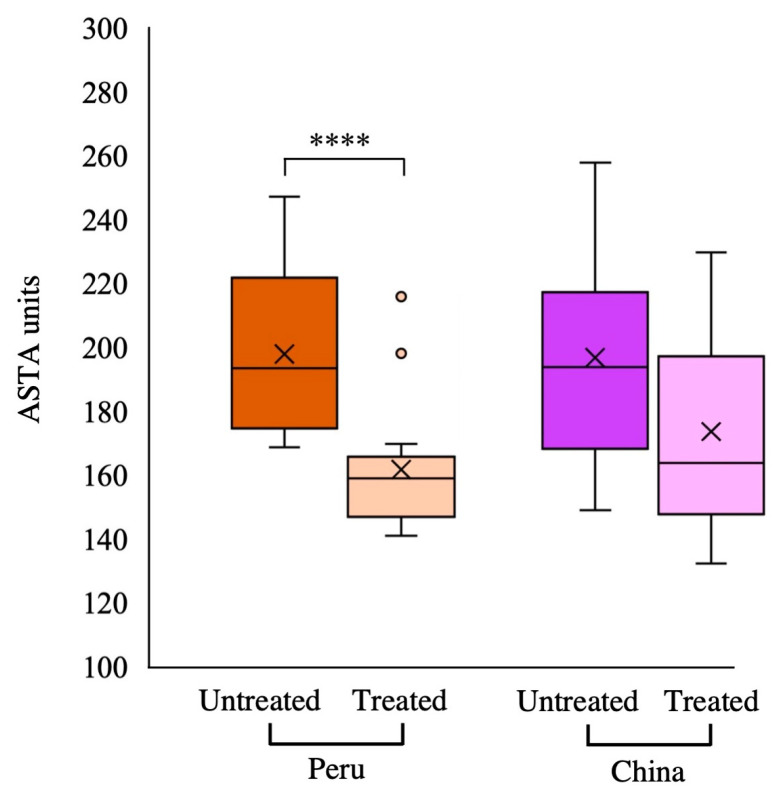
ASTA values of Peruvian (orange) and Chinese (purple) origin. Untreated (intense colour) and treated (light colour). **** Indicates significant differences between samples within the same origin *p* < 0.001).

The colour measurement of paprika is crucial to establishing its quality in the food industry since it is related to the pigment content and the extent to which these pigments have degraded. The colour parameters of the untreated and treated paprika samples were analysed by reflectance using the CIEL* a* b* colour space (Figure 4) and spectrophotometrically by the ASTA units (Figure 5). Significant differences between the samples according to their origin (*p* < 0.01, Table 1) were observed only for L* and H° values. The paprika samples from China showed greater lightness and a lower H° value (Figure 4), indicative of a brilliant and intense red colour. This has been related to the higher content of esterified carotenoids found in these samples, which is characteristic of ripened peppers [43]. No significant differences were observed between samples for the remaining colour parameters, mainly due to the high degree of variability among all samples analysed within each group (*n* = 15). Besides, the samples exhibited a content of esterified carotenoids of around 30% of the total carotenoid content, which has been identified as more resilient to thermal treatment. This may be a contributing factor to the undetected alterations in colour parameters following the heat treatment [44].

Angle H° values close to 0 indicate a higher reddening and, therefore, a higher carotenoid content. Conversely, the loss of carotenoids would imply an increase in H° values. In the case of samples from China, there was a strong negative correlation between total carotenoid content and angle H° (r = −0.741 for untreated samples and r = −0.764 for treated samples, *p* < 0.01). On the other hand, in samples of Peruvian origin, the correlation between total carotenoids and H° values was moderate in untreated samples (r = −0.503, *p* < 0.01) and very weak in treated samples (r = −0.304, *p* = 0.2).

One of the main characteristics of paprika is its striking red colour, which is provided by carotenoids. As previously discussed, the treatment applied significantly resulted in a loss in the concentration of total carotenoids, which was more pronounced in the Peruvian samples than in the Chinese samples. Therefore, the heat treatment also exerted a more pronounced effect on the colour parameters in the Peruvian samples compared to the Chinese. In the former samples, the parameters L* and a* decreased significantly while the H° angle increased, whereas, in Chinese samples, only the a* parameter significantly decreased. These changes indicated a clear degradation of the red colour in all samples, which was directly related to the carotenoid degradation and responsible for the reddish colour of paprika. Some authors have directly linked the application of high temperatures during the paprika production process to a decrease in the CIEL* a* b* colour parameters [15,62]. The results showed a different behaviour of the colour parameters after the heat treatment depending on the origin. It is noteworthy that the H° value did not show significant differences in Chinese samples after the thermal treatment, indicating that the colour of these samples was more stable. While a slightly significant decrease was observed in a* in Chinese samples, the tone perceived by the consumers will remain largely consistent. This discrepancy in colour stability due to the origin may be attributed to the different content of individual and total carotenoids in the samples. The Chinese samples had a 30% and 50% higher content of all carotenoids in the untreated and treated samples, respectively, but this could also be due to the higher content of esterified carotenoids that may contribute to enhanced stability.

Furthermore, during the crushing stage of the paprika production process, the cell walls of the pepper are broken, leaving carotenoids unprotected during the subsequent drying phase. Besides, the presence of reducing sugars and amino acids can trigger the degradation of carotenoids through Maillard reactions, resulting in the formation of melanoidins [63,64]. These compounds are responsible for the characteristic brown colour, which is evidenced by a decrease in brightness (parameter L*) and a decrease in red hue (parameter a*), as observed in the results of our study. Some authors have observed that Maillard reactions are influenced by various factors, including the drying method and the intensity of the heat treatment used. The greater the intensity of the heat treatment, the greater the browning [8,65,66]. Furthermore, it is evident that colour loss is a significant issue not only during the production of paprika but also during storage [67].

The ASTA units, representing the extractable colour according to the American Spice Trade Association, are directly correlated to the carotenoid content [68] and are the most prevalent method for classifying paprika quality in the food industry. The ASTA units did not exhibit any significant differences according to the origin in the untreated paprika samples, but they were significantly affected by the thermal treatment (Table 1). In agreement with the findings of our study, Vega-Galvez et al. [68] showed that the use of high temperatures during the production of paprika resulted in a reduction in ASTA values. The ASTA units for both origins (Peru and China) were higher in untreated samples (198.1 and 196.2, respectively) than in treated samples (161.9 and 172.9, respectively) (Figure 5), indicating significant differences in Peruvian paprikas after heating but not in Chinese samples. This finding may be directly related to a decrease of about 50% in the total carotenoid content following the heat treatment in Peruvian samples, whereas in the samples from China, the losses were about 26% of the total carotenoids. In addition, these results agree with the values of the colour parameters since the paprika samples from Peru exhibited a notable variation of L*, a* and H°.

We were able to verify that differences in the total carotenoid content between samples would not always be reflected in colour differences by analysing ASTA values, the internationally and globally recognised method for determining the quality of paprika. In the present study, a 50% reduction in the carotenoid content with the thermal treatment was necessary to observe differences in the ASTA values between the untreated and treated samples, as observed in the Peruvian samples. However, the reduction of 26% of the total carotenoids in the Chinese samples was not reflected in terms of ASTA values.

In addition, Pearson’s correlation analysis showed a moderate to strong correlation between ASTA units and total carotenoid contents. The correlation coefficients for the untreated samples were r = 0.28 (*p* = 0.33) for Peru and r = 0.86 (*p* < 0.001) for China. For the treated samples, only those of Chinese origin showed a correlation with a value of 0.76 (*p* < 0.01). Therefore, the ASTA values provide general information about the pigments of paprika, but this method does not allow for the measurement or estimation of the degradation of the carotenoids during processing and storage since an intensive change in the content of these bioactive compounds appears to be necessary. Hence, the real degradation of the carotenoids can only be obtained by analysing the samples by HPLC-DAD.

## 4. Conclusions

The observed differences in the content of carotenoids, colour parameters, and the antioxidant capacity of the samples depend on the origin and thermal treatment. Both factors independently affected the physicochemical properties studied, being the contents of individual and total carotenoids more influenced by origin and heat treatment than colour parameters. This may be due to the higher sensitivity of the HPLC-DAD instrument compared to the colourimeter or spectrophotometer. Differences between the origins may be attributed to variations in the esterified carotenoid content among the samples, which may confer higher oxidative and thermal stability to Chinese samples. The colour measurements (CIEL*a*b* and ASTA values) were insufficient to establish differences in the carotenoid contents of the samples, which were only detected by HPLC-DAD. The findings of this study provide valuable insights into the production and commercialisation of paprika by the food industry, emphasising the necessity for tailored approaches, according to the carotenoid composition, for the preservation of quality, nutritional and antioxidant properties of paprika. Further research in this area may contribute to the refinement of processing techniques for the improvement of colour, carotenoid content, and antioxidant capacity retention in paprika.

## Figures and Tables

**Table 1:** Results of two-way ANOVA.

Analyses	Origin	Treatment	O × T
α-carotene	<0.001 *	<0.001	0.979
β-carotene	0.002	<0.001	0.204
Esterified carotenoids	<0.001	<0.001	0.74
Other carotenoids	<0.001	<0.001	0.485
Total carotenoids	<0.001	<0.001	0.537
FRAP	<0.001	<0.001	<0.001
ABTS^•+^	0.608	<0.001	0.787
L	0.003	0.188	0.745
a*	0.769	<0.001	0.024
b*	0.366	0.461	0.29
C*	0.307	0.012	0.749
H°	0.02	<0.001	0.016
ASTA value	0.557	<0.001	0.409

* *p* values.

**Table 2:** Content of α-carotene, β-carotene, esterified carotenoids, and other carotenoids expressed in mg carotenoids/g sample.

Sample	α-Carotene	β-Carotene	Esterified Carotenoids	Other Carotenoids
Peru untreated	12.2 ± 3.4 ^ΩΩΩ^	20.7 ± 6.5	18 ± 4.2 ^ΩΩΩ^	15.2 ± 4.6 ^Ω^
Peru treated	6.4 ± 3.3 ^ΩΩΩ^	10.6 ± 4.9 ^ΩΩ^	9.8 ± 5.3 ^ΩΩΩ^	7.6 ± 5.2 ^ΩΩ^
Statistics	**	***	***	**
China untreated	21 ± 6.9	23.9 ± 6.8	28.3 ± 6.5	21.2 ± 7.2
China treated	15.1 ± 3.7	17.8 ± 4.6	21 ± 4	15.8 ± 5.2
Statistics	**	*	**	-

* Indicates significant differences between samples within the same origin; * (*p* < 0.05), ** (*p* < 0.01), *** (*p* < 0.001). ^Ω^ Indicates significant differences between samples from different origins and the same set point (untreated or treated); ^Ω^ (*p* < 0.05), ^ΩΩ^ (*p* < 0.01), ^ΩΩΩ^ (*p* < 0.001).

## Data Availability

The dataset is available upon request from the authors.
